# Double Peptide-Functionalized Carboxymethyl Chitosan-Coated Liposomes Loaded with Dexamethasone as a Potential Strategy for Active Targeting Drug Delivery

**DOI:** 10.3390/ijms26030922

**Published:** 2025-01-22

**Authors:** Loredana Iftode, Anca Niculina Cadinoiu, Delia Mihaela Raţă, Leonard Ionuț Atanase, Gabriela Vochiţa, Luminița Rădulescu, Marcel Popa, Daniela Gherghel

**Affiliations:** 1 Faculty of Medicine, “Grigore T. Popa” University of Medicine and Pharmacy, 700115 Iasi, Romania; iftodeloredana@yahoo.com (L.I.); lmradulescu@yahoo.com (L.R.); 2 “Cristofor Simionescu” Faculty of Chemical Engineering and Environmental Protection, “Gheorghe Asachi” Technical University, 700050 Iasi, Romania; 3 Department of Biomaterials, Faculty of Medical Dentistry, “Apollonia” University of Iasi, 700511 Iasi, Romania; iureadeliamihaela@yahoo.com (D.M.R.); leonard.atanase@yahoo.com (L.I.A.); 4 Academy of Romanian Scientists, 050044 Bucharest, Romania; 5 Institute of Biological Research Iasi, Branch of NIRDBS—National Institute of Research and Development of Biological Sciences Bucharest, 700107 Iasi, Romania; gabrielacapraru@yahoo.com (G.V.); daniela_gherghel@yahoo.com (D.G.)

**Keywords:** liposomes, carboxylated chitosan, peptides

## Abstract

Liposomes are intensively used as nanocarriers for biology, biochemistry, medicine, and in the cosmetics industry and their non-toxic and biocompatible nature makes these vesicles attractive systems for biomedical applications. Moreover, the conjugation of specific ligands to liposomes increases their cellular uptake and therapeutic efficiency. Considering these aspects, the aim of the present study was to obtain new formulations of cationic liposomes coated with dual-peptide functionalized carboxymethyl chitosan (CMCS) for the treatment of inner ear diseases. In order to achieve efficient active targeting and ensuring a high efficacy of the treatment, CMCS was functionalized with Tet1 peptide, to target specific ear cells, and TAT peptide, to ensure cellular penetration. Furthermore, dexamethasone phosphate was loaded as a model drug for the treatment of ear inflammation. The infrared spectroscopy confirmed the functionalization of CMCS with the two specific peptides. The mean diameter of the uncovered liposomes varied between 167 and 198 nm whereas the CMCS-coated liposomes ranged from 179 to 202 nm. TEM analysis showed the spherical shape and unilamellar structure of liposomes. The release efficiency of dexamethasone phosphate after 24 h from the uncoated liposomes was between 37 and 40% and it appeared that the coated liposomes modulated this release. The obtained results demonstrated that the liposomes are hemocompatible since, for a tested concentration of 100 µg/mL, the liposome suspension had a lysis of erythrocytes lower than 2.5% after 180 min of incubation. In addition, the peptide-functionalized CMCS-coated liposomes induced a non-significant effect on the viability of normal V79-4 cells after 48 h, at the highest doses. Values of 71.31% were recorded (CLCP-1), 77.28% (CLCP-2) and 74.36% (CLCP-3), correlated with cytotoxic effects of 28.69%, 22.72%, and 25.64%.

## 1. Introduction

The recent progress of medicine is not only the consequence of our greater understanding of biological processes, but it is especially due to the way in which technology makes it possible to disseminate and exploit the obtained results. Nanomedicine, as the medical application of nanotechnology, lies at the interface of three major disciplines: biology, physics, and chemistry, and it is based on the use of nanoparticles (NPs) for the diagnosis and treatment of different diseases [1]. In diagnosis, nanostructures can be used for both in vitro and in vivo diagnosis whereas in therapy, the NPs are used for the vectorization or delivery of certain active principles by passive or active targeting [2].

The developed NPs must be biocompatible, preferably biodegradable, stable, and have a long in vivo circulation time. In view of its application on an industrial scale, it is important that the manufacturing process of these NPs is simple with a low cost price.

There are a wide variety of NPs ranging from inorganic to organic particles, including liposomes or polymeric NPs, such as nanospheres and nanocapsules, dendrimeres, micelles, and polymersomes [3,4,5]. If the choice of the core of the particles is essential with regard to the protection and the sustained release of the active principles, the control of the surface is just as important. It is in fact its surface properties that allow the particle to convey the loaded cargo to the targeted area [6,7,8,9].

Liposomes are intensively used as nanocarriers for biology, biochemistry, medicine, and the cosmetics industry. Their non-toxic and biocompatible nature makes these vesicles attractive systems for in vivo applications. However, liposomes also have the following limitations: moderate stability, delicate production, and non-specific targeting [10]. Upon entering the bloodstream, liposomes are rapidly surrounded by plasma proteins or other biomolecules [11], forming a protein shell, called a “protein corona”, and the newly formed complex can be very different from pure liposomes [12,13,14,15,16]. In addition, the functionalization of liposomes with different ligands for better targeting and their nature have a major impact on the formed protein coronas, influencing the fate and transport of liposomes [17,18,19,20]. Therefore, great importance is afforded to understanding the mechanism by which peptide-functionalized liposomes interact with plasma proteins to design liposomes with high targeting and good in vivo immunocompatibility. Among different types of liposomes, cationic liposomes showed promising in vitro results as drug delivery systems [21,22,23]. One of the most used cationic lipids is 1,2 dioleoyloxy-3-(trimethylammonio) propane (DOTAP) [24,25,26,27]. Although these systems present numerous advantages for drug administration, important disadvantages should not be overlooked, as they present real challenges for clinical translation. Cationic liposomes have often been studied for gene delivery and many studies have shown that these systems can cause toxicity in phagocytic cells [28,29]. Some researchers have correlated different properties, such as surface charge, size, or pegylation with the immunogenic responses of cationic liposomes [30,31]. In order to improve the stability of these drug delivery systems, the surface of cationic liposomes can be coated with polymers, such as chitosan [32] or carboxymethyl chitosan (CMCS) [33] which are biodegradable, biocompatible, and non-toxic. Generally, the coating of liposomes with polymers will induce an increase in the sizes and a change of zeta potential (ZP) values [34], but it can, on the other hand, improve pharmacokinetics, biodistribution, or toxicity [35].

The specific targeting of different sites of the human body can be achieved by using various ligands, such as aptamers, peptides, and antibodies [36,37]. Peptides, generally having less than 30 amino acids in their structure, have emerged as successful ligands in active targeting drug delivery due to their higher tumor penetrating properties as compared to conventional antibodies, facile synthesis process, higher selectivity than aptamers, non-immunogenic properties, and easy chemical modification [38,39]. The conjugation of specific ligands to liposomes, carried out directly to phospholipids and/or polymers, increases their cellular uptake and therapeutic efficiency [39].

D’Avanzo et al. obtained LinTT1 peptide-functionalized liposomes co-loaded with doxorubicin and sorafenib for the treatment of triple negative cancer [40]. An internalization of 50% in M2 primary human macrophages was observed whereas the other 50% of the functionalized liposomes were adsorbed at the cell surface. In another study, Giofrè et al. investigated dual-surface functionalized liposomes in order to cross the blood–brain barrier (BBB) [41]. A modified peptide, used an enhancer to cross the BBB, and a matrix metalloproteinase as an enzymatic drug release trigger, were used for the functionalization. It was demonstrated on an in vitro model, that these peptide-functionalized liposomes can cross the BBB. Peptide-functionalized liposomes were also used for arthritis therapy administrated by a subcutaneous route [42]. It appeared that liposomes injected into arthritic rats showed preferential homing to arthritic joints.

An interesting study explored the functionalization of liposomes with a pH-sensitive peptide suitable for targeting B16F1o melanoma cells by using two strategies. On one hand the peptide was linked to the DSPE-PEG2000-Mal and on the other the peptide was modified with a C18 stearyl chain and used in the composition of the phospholipid bilayer [43]. The latest method induced a higher cellular uptake efficiency which suggests that the functionalization method has a high importance on the targeting efficiency. In a very recent study, NFL peptide-functionalized liposomes demonstrated a consequent and systematic increased uptake into Rat F98 glioblastoma cells [44].

Cell-penetrating peptides (CPPs), such as TAT peptides, were used for the preparation of active-targeted drug-loaded liposomes which might have a main advantage of a lower toxicity during therapy [17,45,46,47].

Inner ear diseases have known several types of treatments over time, starting from systemic administration, continuing with intratympanic administration and recently reaching the administration of nanosystems designed to deliver drugs directly to the inner ear over a sustained period of time [8]. Anti-inflammatory drugs are most often used, which, unfortunately, are quickly eliminated due to the short half-life in the cochlea. Dexamethasone is known to be one of these active principles that suppress inflammation and has been widely used as a potential therapy for treating inner ear pathologies [48,49]. Even though several treatment options are available, current approaches do not ensure sufficient efficacy and safety. In the case of systemic therapy, subtherapeutic concentrations of drugs in the inner ear and adverse effects were noted [50], while in the case of intratympanic injections, elimination of the drug through the Eustachian tube, inconsistent diffusion rate in the inner ear, but also an increased risk of damage to the middle ear structures was observed [51]. Injuries to the inner ear were also noted in the direct microinjection of drugs through the cochleostomy [52]. Due to these disadvantages, researchers in collaboration with otorhinolaryngologists have tried, in the last decades, using several drug release systems, such as polymeric nanoparticles, liposomes, cubosomes, lipid-based nanocarriers, or inorganic nanoparticles [53,54], to treat various diseases of the inner ear.

The aim of this study was to obtain, in a first step, a series of cationic liposomes based on DOTAP which were further coated with peptide-functionalized carboxylated chitosan. For functionalization, two peptides were chosen, TAT, a cell-penetrating peptide, and Tet1, a peptide that targets specific cells in the inner ear. The obtained nanocarriers were characterized from several points of view. Thus, the morphological characteristics, the capacity to load/release a hydrophilic active principle, the in vitro hemolytic potential, and the cytotoxic effect on fibroblast cells were evaluated. Dexamethasone phosphate was loaded as the model hydrophilic active principle, as it is often used in the treatment of inner ear diseases [55]. Considering the challenges connected to liposomal systems, it is necessary to mention that the main aim of the study was to investigate the short-term in vitro effects, in order to subsequently follow their in vivo behavior.

## 2. Results

### 2.1. Structural Characteristics

**Figure 1:**
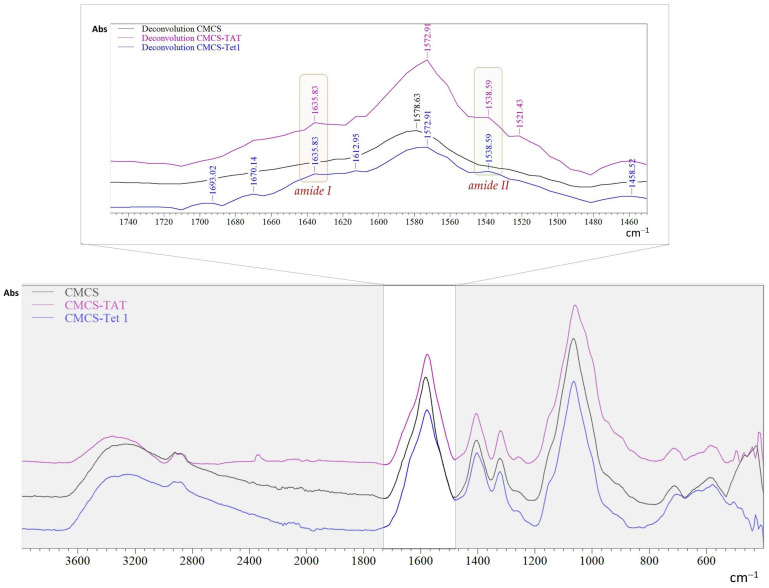
FTIR spectral profiles of CMCS and CMCS functionalized with TAT (CMCS-TAT) and Tet-1 peptides (CMCS-Tet1).

Infrared spectroscopy (FTIR) was used to confirm the functionalization of carboxylated chitosan with the two peptides. The FTIR spectra for CMCS and for TET1- and TAT-functionalized CMCS are presented in Figure 1. A spectral deconvolution in the range of 1450–1750 cm^−1^ was performed to highlight the presence of specific bands for amides in the peptide structure. The specific absorption band for amide I was found at 1635 cm^−1^ whereas the specific absorption band for amide II was found at 1538 cm^−1^ [56,57].

The functionalization of CMCS with the two peptides was also analyzed using ^1^H NMR spectroscopy. The results obtained are presented in Supplementary Materials, Figure S1.

### 2.2. Characterization of Peptide-Functionalized Liposomes

Both dexamethasone-loaded cationic liposomes (CLD-1, CLD-2, and CLD-3) and drug-loaded samples coated with a mixture of non-functionalized and peptide-functionalized CMCS (CLDCP-1, CLDCP-2, CLDCP-3) were characterized from several points of view, as follows.

The intensity-weighted hydrodynamic diameter of the uncovered liposomes ranged between 167 and 198 nm, as can be seen in Table 1. The smallest diameter was noticed for sample CLD-3, which had the highest percentage of cholesterol in its composition. Moreover, the coating with CMCS led to an increase in the average diameter, as expected. The average size of CMCS-coated liposomes varied from 179 to 202 nm.

**Figure 2:**
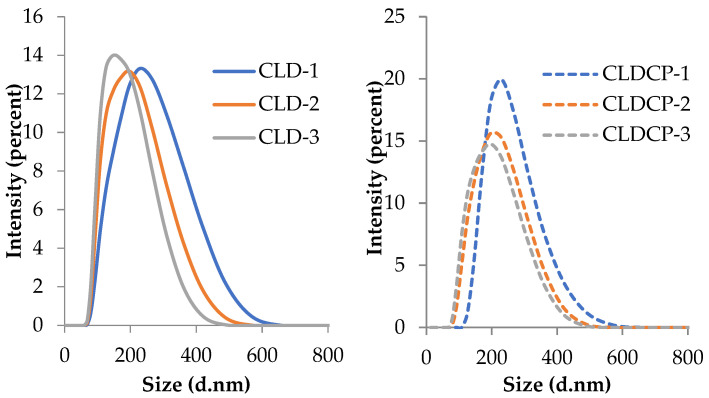
The intensity-weighted distribution of cationic liposomes (CLD-1, CLD-2, CLD-3) and cationic liposomes coated with CMCS (CLDCP-1, CLDCP-2, CLDCP-3) in ultrapure water.

Figure 2 shows the intensity-weighted distribution of uncoated cationic liposomes and CMCS-coated cationic liposomes. As can be seen, the distribution curves are monomodal and right-skewed or positive, indicating that a small proportion of the particle population is larger than 400–500 nm in size. However, this proportion is very low, a fact that is reflected in the PDI values.

The zeta potential was evaluated to determine both the stability of the encapsulation systems and whether CMCS coating had occurred. The zeta potential values for the uncoated CLD liposomes were found to be +10.71, +24.91, and +24.6 mV, respectively (Table 1). The positive value of the zeta potential is attributed to the presence of cationic lipid in the structure of the liposomes. After coating, CMCS binds to the positively charged surface of the liposomes, thus changing the surface charge of the liposomes towards the negative due to the carboxylic groups present in CMCS.

#### 2.2.1. The Morphology of Liposomes

**Figure 3:**
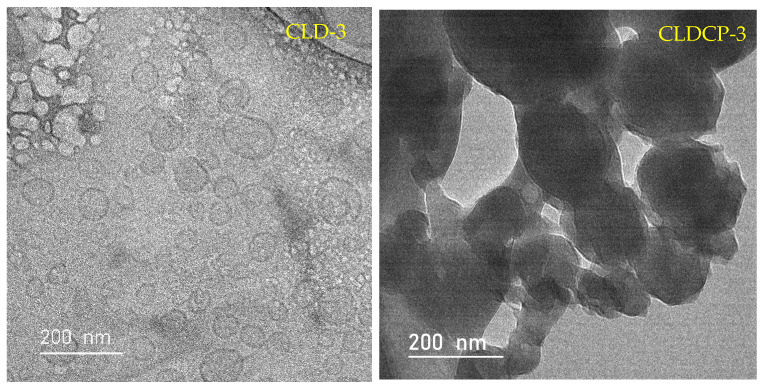
TEM micrographs of CLD-3 (uncoated) obtained at an acceleration voltage of 200 kV with a magnification of 25,000 and CLDCP-3 (coated) samples obtained at an acceleration voltage of 40 kV with a magnification of 25,000.

TEM analysis showed that liposomes are spherical in shape and most of them have a unilamellar structure, as can be seen in Figure 3. No aggregates were observed in the case of the uncoated liposomes and the results can be correlated with the results obtained by DLS. For the CLDCP-3 sample of liposomes covered with CMCS, a slight agglomeration of the vesicles can be observed, but also an obstruction on their surface due to the interaction between the cationic lipid and CMCS.

#### 2.2.2. The Encapsulation Efficiency

The loading efficiency of the dexamethasone phosphate (Dex-P) in uncoated liposomes was between 21 and 26% (Table 2). This percentage is the amount of drug found in the liposomes after their destruction compared to the amount of drug in the solution used to hydrate the lipidic film. The drug loading efficiency for CMCS-coated liposomes was determined after the coating step by subtracting from the amount of drug loaded in the uncoated liposomes from the amount of drug found in the supernatant after the coating step.

#### 2.2.3. The Drug Release Tests

The in vitro drug release tests from uncoated liposomes and CMCS-coated liposomes were determined at 37 °C, in phosphate buffered saline (PBS) at pH 7.4, to simulate conditions in human body, related to pH [58].

**Figure 4:**
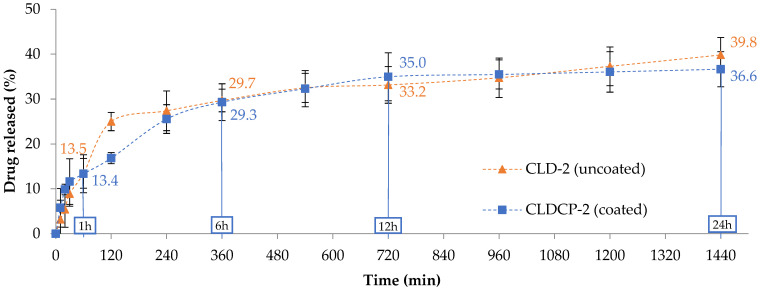
The percentage of cumulative drug released from cationic liposomes and cationic liposomes coated with CMCS for 24 h, highlighting the released percentage at specific time intervals (1 h, 6 h, 12 h, and 24 h). Data presented as mean ± SD, n = 3.

The release efficiency of the anti-inflammatory drug from the uncoated liposomes (Table 2) was slowed down by the lipid matrix. Thus, release degrees between 37 and 40% were recorded after 24 h in a temperature- and pH-controlled environment. To help liposomes target the inner ear, they were coated with a mixture of CMCS and peptide-functionalized CMCS. The drug release efficiency from the coated liposomes showed a decrease of 3–5%, after 24 h, compared to uncoated liposomes (Table 2), probably due to the CMCS coating that provides extra protection to the lipid vesicles. For the release tests, drug-loaded liposome samples as well as free drug, in an amount similar to that loaded in the liposomes, were placed in cellulose dialysis tubes and sealed well, before being immersed in PBS solution at 37 °C in a water bath under continuous stirring. Dexamethasone phosphate concentrations in the receptor medium were determined by UV-Vis spectroscopy at 242 nm. This dynamic dialysis method is a method often used to study the release kinetics from nanocarriers. Free drug, at a concentration equal to that loaded in liposomes, diffuses through the dialysis membrane to the “sink” receptor compartment in nearly 4 h (Figure S2, in the Supplementary Materials). In the case of drug-loaded liposomes, the drug must cross two barriers, the lipid membrane and the dialysis membrane, to reach the receptor compartment. Figure 4 shows the release curves of dexamethasone from two typical formulations, i.e., uncoated liposomes (CLD-2) and CMCS-coated liposomes (CLDCP-2). Only these two formulations were chosen as typical examples because the other four have a similar behavior. The plotted release curves show two phases, an initial burst release in the first 30 min followed by a sustained release of the drug over the monitored time period. Thus after 24 h, it can be observed that the drug was released in a proportion of approximately 40% in the case of the uncoated liposomes and in a proportion of approximately 37% in the case of the coated liposomes.

#### 2.2.4. The Stability Tests

**Figure 5:**
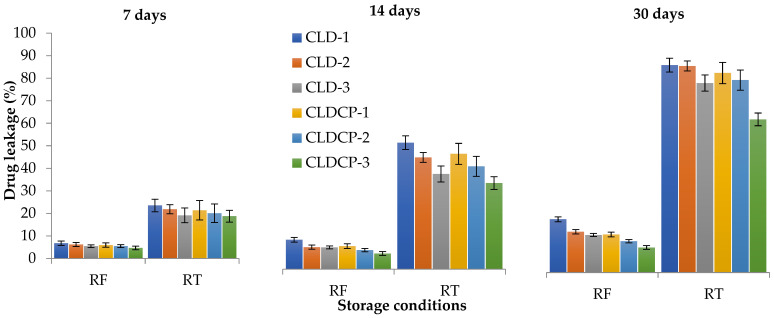
Drug leakage from samples stored at two different temperatures, 4 °C (RF) and 25 ± 2 °C (RT) as a function of time. Data presented as mean ± SD, n = 3.

Liposomes can be physically unstable during storage, leading to leakage of encapsulated drugs [59]. The stability of liposomes can lead to degradation of the active ingredient, which can lose potency or undergo undesirable chemical reactions, leading to reduced therapeutic efficacy [60]. The stability of cationic liposomes, coated and uncoated, was studied for 30 days in PBS solution with pH 7.4 at two different temperatures, 4 °C (refrigerator) and 25 ± 2 °C (room temperature). Drug leakage after 30 days ranged between 10 and 21% for samples kept in the refrigerator and between 62 and 84% for samples kept at room temperature, as can be seen in Figure 5 which indicates that their stability is higher at lower temperatures.

#### 2.2.5. The Hemolytic Potential

**Figure 6:**
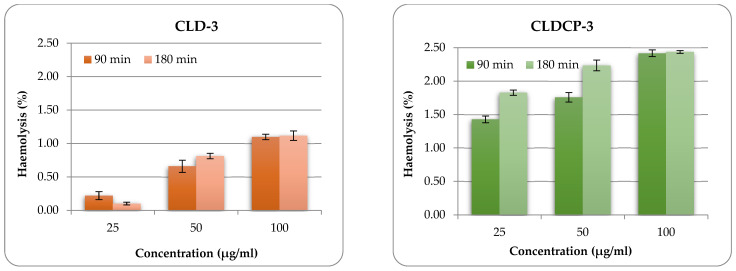
Hemolysis percentage after 90 and 180 min exposure to CLD-3 and CLDCP-3 liposome samples. Data presented as mean ± SD, n = 3.

The in vitro hemolytic potential of liposomes was evaluated using a spectrophotometric method. This method can determine the amount of oxyhemoglobin released as a result of damage to the membranes of red blood cells in contact with the liposome’s suspension. The obtained results are presented in Figure 6.

Typical liposomal samples were also evaluated in terms of hemolytic properties because they could also be administered intravenously. The liposomes obtained (with and without CMCS coating) were incubated with red blood cells at three different concentrations. The oxyhemoglobin released was measured at 2 different time intervals (90 min and 180 min). From Figure 6 and Figure S3, it can be seen that the hemolytic percentage increases with the increase in the concentration of liposomes, as expected. Another observation that emerges from Figure 6 is that liposomes coated with CMCS and functionalized with peptides present a higher degree of hemolysis compared to uncoated liposomes, this was also observed in the case of other particulate systems functionalized with peptides obtained by our research group [61]. However, even at the highest concentration tested (100 µg/mL), the liposome suspension did not produce a lysis of erythrocytes greater than 3% after 180 min of incubation (Figure 6 and Figure S3). This behavior leads us to the conclusion that this type of system can be included in the category of non-hemolytic materials.

### 2.3. Assessment of the In Vitro Cytotoxicity

For testing the in vitro biocompatibility of the newly synthesized nanocarriers, normal V79-4 cell cultures (fibroblasts) were chosen using a wide range of doses between 3.125 and 25 µg/mL. Morphological studies were also performed to confirm the degree of interference of the liposomes with the cells. The systems without loaded drug were tested, coded as follows: CL-1, CL-2, and CL-3 (uncoated liposomes); CLC-1, CLC-2, and CLC-3 (liposomes coated with non-functionalized CMCS), respectively; and CLCP-1, CLCP-2, and CLCP-3 (liposomes coated with a mixture of non-functionalized and peptide-functionalized CMCS).

**Figure 7:**
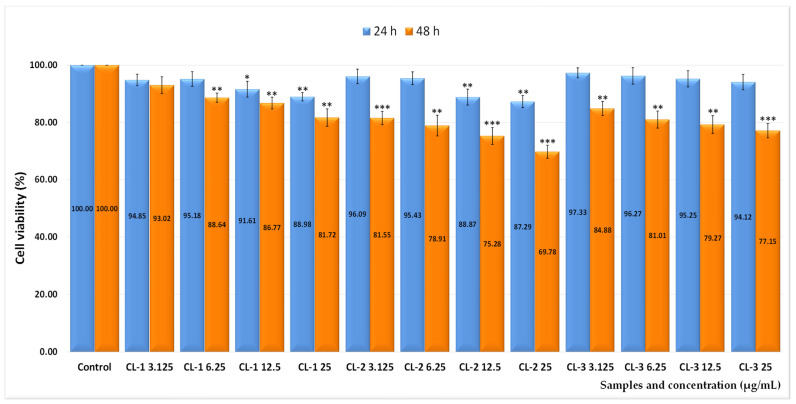
Effect of 24 and 48 h treatment with different concentrations of CL-1, CL-2, and CL-3 cationic liposomes on the viability of normal V79-4 cell cultures. Error bars indicate standard error of the mean (SEM), n = 3. * *p* < 0.05, ** *p* < 0.01, *** *p* < 0.001.

Thus, the 24 h treatment, for all cationic liposomes induced an insignificant effect on the viability of V79-4 normal cells, being practically non-cytotoxic even at the highest dose tested. As can be seen from Figure 7, the 48 h treatment caused, at the highest doses, low decreases in cell viability for CL-1 and CL-3 such as: 81.72% and 77.15%, respectively, corresponding to cytotoxic effects of 18.28% and 22.85%, respectively, while CL-2 interfered most strongly with cell viability (69.78%), inducing a more pronounced cytotoxic action of 30.22%.

**Figure 8:**
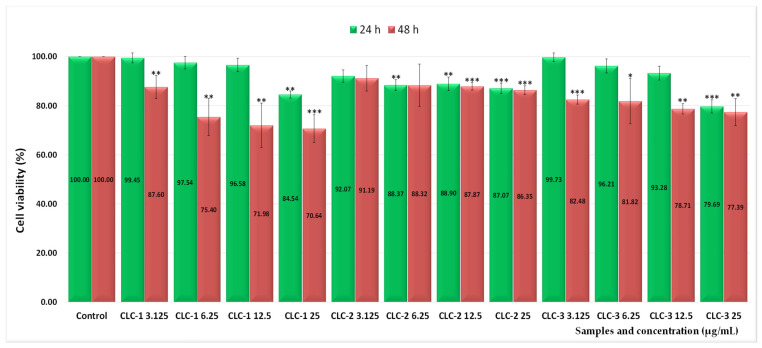
Effect of 24 and 48 h treatment with different concentrations of CLC-1, CLC-2, and CLC-3 cationic liposomes coated with CMCS on the viability of normal V79-4 cell cultures. Error bars indicate standard error of the mean (SEM), n = 3. * *p* < 0.05, ** *p* < 0.01, *** *p* < 0.001.

Furthermore, the effect of CMCS coating on cell viability was investigated. Thus, as can be seen from Figure 8, the 48 h treatment with cationic liposomes coated with CMCS, at 25 µg/mL, resulted in cell viability values of 70.64% (CLC-1), 86.35% (CLC-2), and 77.39% (CLC-3), corresponding to low cytotoxicity values, of 29.36%, 13.65%, and 22.61%, respectively. 

**Figure 9:**
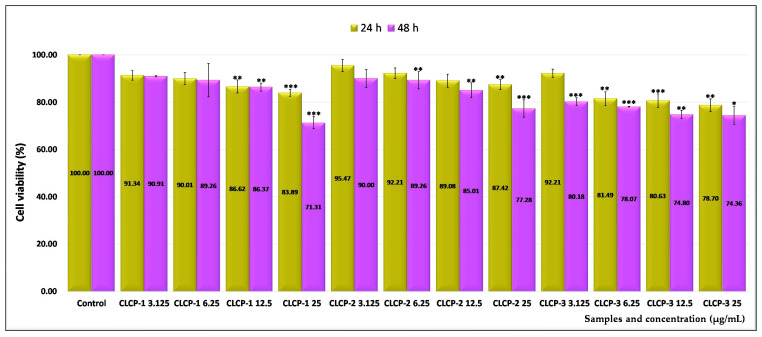
Effect of 24 and 48 h treatment with different concentrations of CLCP-1, CLCP-2, and CLCP-3 cationic liposomes coated with a mixture of non-functionalized and peptide-functionalized CMCS on the viability of normal V79-4 cell cultures. Error bars indicate standard error of the mean (SEM), n = 3. * *p* < 0.05, ** *p* < 0.01, *** *p* < 0.001.

Further, the effect of cationic liposomes coated with a mixture of non-functionalized and peptide-functionalized CMCS on the viability of normal V79-4 cells was also investigated. The treatment with these samples for 48 h led to a moderate interference with cell viability being recorded. At the highest doses, the values of 71.31% (CLCP-1), 77.28% (CLCP-2), and 74.36% (CLCP-3), correlated with cytotoxic effects of 28.69%, 22.72%, and 25.64%, as results from the analysis of Figure 9.

### 2.4. The Half-Maximal Inhibitory Concentration (IC50) Values

The IC50 values were calculated based on the results of the MTT cell viability test performed on the V79-4 cell line and are shown in Table 3. These values, corresponding to the 24 h treatment, ranged from 40.70 µg/mL (CLCP-3) to 190.39 µg/mL (CL-3), whereas, after the 48 h treatment, the IC50 values decreased, being in the range of 31.32 µg/mL (CL-2) to 63.90 µg/mL (CLC-2), due to the longer contact with the cells.

### 2.5. Morphology Assay

According to the photos (Figures S4–S12), at low doses, minor morphological changes were recorded. On the other hand, the highest concentrations of CLCP-3 application induced the blocking of normal cell functionality due to the aggregation of this type of CMCS-coated liposomes.

In accordance with international standards [62,63], it can be concluded that cationic liposomes belong to the categories of non-cytotoxic (CL-1) and weakly cytotoxic nanostructures (CL-2 and CL-3). The liposomes coated with CMCS fit into the non-cytotoxic (CLC-2) or weakly cytotoxic category (CLC-1 and CLC-3). Lastly, cationic liposomes coated with a mixture of non-functionalized and peptide-functionalized CMCS expressed weakly cytotoxic action (CLCP-1, CLCP-2, and CLCP-3). These results obtained from the MTT viability tests were also confirmed by cell morphology tests, in which changes regarding cellular adhesion are observed in a dose- and time-dependent manner.

## 3. Materials and Methods

### 3.1. Materials

Egg yolk phospholipids (Lipoid E PC S) (Phosphatidylcholine content: ≥96%) (EPC) and 1,2-Dioleoyloxy-3-trimethylammonium-propane chloride (Lipoid DOTAP) (DOTAP) were purchased from Phospholipid GmbH, Köln, Germany. Carboxymethyl chitosan (CMCS) (with a degree of carboxylation of 80%) was purchased from Sigma Aldrich, St. Louis, MO, USA. Cholesterol (CHOL), dexamethasone phosphate disodium salt (DexP), and fluorescein disodium salt (Flu) were purchased from Alfa Aeser (ThermoFisher, Waltham, MA, USA), Karlsruhe, Germany. Chloroform and Triton X were obtained from VWR International LLC, Radnor, PA, USA. TAT (sequence RKKRRQRRR) and Tet1 (sequence HLNILSTLWKYR) peptides were purchased from Eurogentec SA, Liege, Belgium. The fibroblasts cell line and DMEM were purchased from ATCC, Manassas, Virginia, USA and the other necessary supplies for in vitro cytotoxicity assay were purchased from Thermo Fisher Scientific (Waltham, MA, USA).

### 3.2. Preparation Methods

#### 3.2.1. Functionalization of CMCS with the Peptides

**Figure 10:**
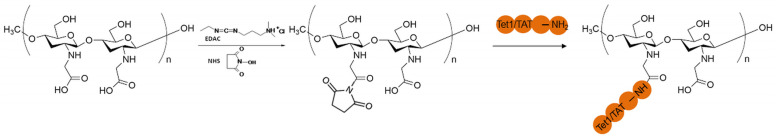
Schematic representation of carboxylated chitosan functionalization with TAT-NH_2_/Tet1-NH_2_peptides.

Chitosan, in the form of a carboxylate derivative, was functionalized by amidation with TAT (sequence RKKRRQRRR) (P1) and Tet1 (sequence HLNILSTLWKYR) (P2) peptides via carbodiimide activation, in the presence of the 1-Ethyl-3-(3-Dimethylaminopropyl)carbodiimide (EDAC) and N-Hydroxysuccinimide (NHS), according to Figure 10.

Briefly, 0.375 mmol of CMCS were dissolved in 15 mL double-distilled water and 0.00375 mmol of TAT-NH_2_ were dissolved in 10 mL double-distilled water, the molar ratio between CMCS and TAT-NH_2_ being 100/1. In total, 40 mL of 0.4 mM EDAC solution and 40 mL of 0.1 mM NHS solution were also prepared separately. The EDAC, NHS, and CMCS solutions were mixed for 30 min at room temperature, and then the TAT-NH_2_ solution was added. The reaction was carried out at room temperature under gentle stirring in the dark for 24 h. The product was purified by dialysis for 24 h, the distilled water being changed 6 times, and finally the CMCS-TAT conjugate was recovered by lyophilization during 24 h. The same protocol and the same molar ratio between the polymer and the peptide were also used for the Tet-1 peptide.

#### 3.2.2. Preparation of Cationic Liposomes

Liposomes are generally obtained from phospholipids, both natural, such as egg or soy phosphatidylcholines, and synthetic, such as dialkyl or trialkyl lipids [64,65]. For this study, a natural phospholipid (egg yolk phospholipids) was used because natural phospholipids, compared to synthetic ones, are available on a wider scale and can be purchased at a lower cost [66]. Cholesterol is often incorporated into liposomes because it modulates membrane permeability and influences fluidity [67,68]. Since our next goal was to coat the liposomes with CMCS and peptide-functionalized CMCS, the liposomes had to be positively charged. As phosphatidylcholine is a zwitterionic molecule with no net charge at neutral pH [69] it was necessary to add a cationic lipid, DOTAP [70], to the composition of the liposomes. This cationic lipid was also used by Yao et al. for obtaining CMCS-coated liposome formulations for sorafenib and siRNA co-delivery [33].

Taking into account the above, the liposomes were obtained from EPC, DOTAP, and CHOL in different molar ratios, using a simple method, i.e., hydration of the thin lipid film, followed by sequential extrusion, which represents the most efficient method of nanosizing liposomes and obtaining unilamellar vesicles with a homogeneous size distribution. Briefly, the lipids were solubilized in chloroform in a 250 mL round-bottom flask. After complete dissolution of the lipids, the chloroform was evaporated under reduced pressure at 160 rpm and 40 °C, using a Heidolph rotary evaporator (Hei-VAP ML), Heidolph Instruments GmbH & Co. KG, Schwabach, Germany). After solvent evaporation, the lipid membrane was further dried under a constant flow of argon for 20 min. The obtained film was then hydrated using 5 mL of phosphate buffered saline (pH = 7.4) at 40 °C. After vigorous stirring, spontaneous formation of multilamellar vesicles occurred. The resulting dispersion was placed in a bath sonicator for 30 min at 40 °C to break up existing aggregates. Finally, the liposome size was reduced by multiple extrusion steps through polycarbonate membranes with a pore size of 0.4 μm (5 times) then 0.2 μm (5 times) using a mini extrusion set from Avanti Polar Lipids, Inc., Alabaster, AL, USA. The experimental program for obtaining liposomes is presented in Table 4.

The dexamethasone phosphate was encapsulated in the liposomes during the preparation process. The drug was solubilized in 5 mL phosphate buffer solution (PBS, pH 7.4). To remove the free drug, the liposome suspension was subjected to a dialysis step using a cellulose tubular membrane (12–14 kDa) against ultrapure water, until dexamethasone was no longer detected spectrophotometrically. More precisely, the obtained liposomes suspension was placed in the tubular membrane, clamped at both ends, and immersed in 200 mL of ultrapure water under gentle magnetic stirring at room temperature. The water was changed every 2 h and the presence of dexamethasone was checked spectrophotometrically.

**Figure 11:**
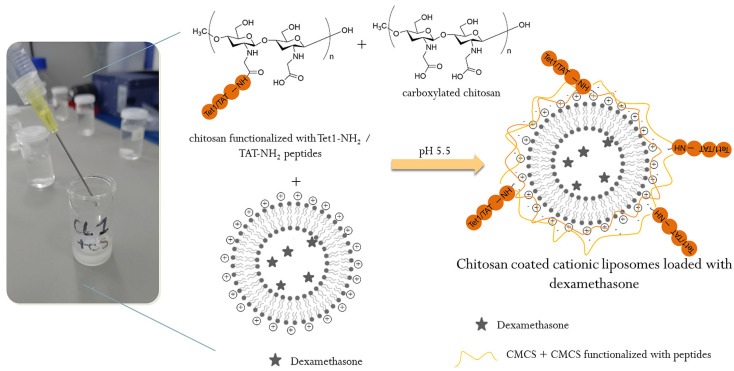
Schematic representation for coating of liposomes with CMCS and peptide-functionalized CMCS.

For the preparation of chitosan-coated liposomes, a mixture of CMCS + CMCS-P1 + CMCS-P2 (2 mL solution, 1% concentration in ultrapure water) was added dropwise to 2 mL liposomes suspension under magnetic stirring at room temperature (Figure 11). The obtained suspension was finally stirred for 2 h in order to accomplish the electrostatic attraction between positive surface changes of liposomes and negative charges of CMCS molecules [71].

### 3.3. Characterization of Peptide-Functionalized CMCS and Peptide-Functionalized Liposomes

#### 3.3.1. Characterization of Peptide-Functionalized CMCS

The structure of the peptide-functionalized CMCS was analyzed by Fourier transform infrared (FT-IR) spectroscopy using a Shimaszu IRSpirit (Schimadzu Europa GmbH, Duisburg, Germany) spectrometer equipped with a single reflection ATR accessory QATR-S. The FT-IR Spectra, in absorbance mode, were recorded at room temperature in the range 400–4000 cm^−1^ with a resolution of 16 cm^−1^. The data were analyzed with LabSolutionsIR software (Version 2.27).

The functionalization of CMCS with the two peptides was also analyzed using ^1^H NMR spectroscopy. The method is presented in the Supplementary Materials.

#### 3.3.2. Characterization of Peptide-Functionalized Liposomes

The intensity mean hydrodynamic diameter and polydispersity index (PDI) of the obtained liposomes were determined by dynamic light scattering (DLS), at 25 °C and at a concentration of 1% (*w*/*v*) in ultrapure water. The zeta potential was measured by laser doppler micro-electrophoresis at 25 °C after dilution in the hydration medium to an appropriate counting rate. Mean diameter and zeta potential were measured using a Zetasizer Nano ZS from Malvern Panalytical Limited, Worcestershire, UK.

Cryo-electron microscopy (cryo-EM) was chosen to determine the morphology of liposomes because it is a precise and direct method to determine the lamellarity, size, shape, and ultrastructure of this type of nanocarrier. An FEI Tecnai G2 F-20 TWIN Cryo-TEM (FEI American Company, Brno, Czech Republic) was used for this analysis. For sample CLD-3, the equipment operated at an acceleration voltage of 200 kV with a magnification of 25,000 while for sample CLDCP-3, it operated at an acceleration voltage of 40 kV.

The encapsulation efficiency of dexamethasone phosphate was determined spectrophotometrically (NanoDrop™ One Microvolume UV-Vis Spectrophotometer, ThermoFisher Scientific, Madison, WI, USA) at 242 nm after prior destruction of the liposomes in the presence of a surfactant (Triton X-100). The calibration curve of DexP in PBS solution was used to calculate the amount of drug encapsulated in the aqueous core of the liposomes. The following equation was used to calculate the DexP encapsulation efficiency (EE_DexP_%):

(1) $${\mathrm{EE}}_{\mathrm{DexP}}\%=\frac{W_{\mathrm{DexP}\;\mathrm{final}}}{W_{\mathrm{DexP}\;\mathrm{initial}}}\times 100$$

where W_DexP final_ is the total amount of the encapsulated drug and W_DexP initial_ is the total amount of drug added initially during the hydration stage.

A PBS solution with a pH of 7.4 was used to assess the release of dexamethasone phosphate from liposomes to simulate, as closely as possible, a physiological environment. This experiment was performed using an Agilent Dissolution Apparatus 708-DS (Agilent Technologies LDA, Bayan Penang, Malaysia) equipped with a Sampling Station, 850-DS. In total, 2 mL of drug-loaded liposomes as well as free drug, in an amount similar to that loaded in liposomes, were introduced in cellulose dialysis tubes, well-sealed, and immersed in 200 mL buffer solution at 37 °C, in a water bath, under continuous stirring. The concentrations of model drug (dexamethasone phosphate) in the receiver medium were determined by UV–Vis spectroscopy using the same equipment and the same parameters as those used for the encapsulation efficiency tests.

The liposomes stability was monitored in PBS solution with pH 7.4 by storing the suspensions in well-sealed containers in the refrigerator (4 °C) and at room temperature (25 ± 2 °C) for one month. For this, 20 μL of liposome suspension was periodically withdrawn and then added to 4 mL of PBS. The amount of drug outside the liposomes was determined spectrophotometrically after centrifugation. Drug leakage was calculated as the cumulative amount of drug leaked over time (found in the supernatant) compared to the amount of drug initially encapsulated in the liposomes. The experiments were performed in triplicate. 

The in vitro hemolytic potential of liposomes was evaluated using the spectrophotometric method [72]. Briefly, for these tests, 5 mL of blood was collected in vacutainer tubes with anticoagulant from a healthy, non-smoking human volunteer after obtaining appropriate informed consent and institutional ethical approval. The collected blood was centrifuged for 5 min at 2000 rpm to separate the erythrocytes and then several washes with normal saline were performed to completely remove the plasma. After purification, 25 mL of suspension was obtained by adding normal saline over the erythrocytes. To test the effect of liposomes on red blood cells, 0.5 mL of suspension with different concentrations of liposomes in saline was added over 0.5 mL of erythrocyte suspension and the following final test concentrations were obtained: 25, 50, and 100 µg liposomes/mL erythrocyte suspension. Positive (100% lysis) and negative (0% lysis) controls were also prepared by adding equal volumes (0.5 mL) of Triton X-100 and standard saline. To closely mimic physiological conditions, samples were incubated for 90 and 180 min at 37 °C, respectively. Samples were gently vortexed every 30 min to avoid sedimentation using a vortex from DLAB Scientific Co., Ltd. After the incubation times mentioned above, samples were centrifuged at 2000 rpm for 5 min and 100 μL of supernatant from each sample was incubated at room temperature for 30 min to allow hemoglobin oxidation. A UV-Vis NanoDrop One spectrophotometer was used to determine the absorbance of oxyhemoglobin in the supernatants at 540 nm. The absorbance value of the sample (Abs_sample_) and the absorbance values of the negative and positive control (Abs_negative control_ and Abs_positive control_, respectively) were used to determine the hemolytic percentage. The experiments were performed in triplicate.

#### 3.3.3. Assessment of the in Vitro Cytotoxicity

For in vitro tests, the healthy cell cultures, respectively, V79-4 fibroblast (ATCC^®^ CCL-93), were used, which were cultured in the Dulbecco’s Modified Growth Medium (DMEM), supplemented with 10% fetal bovine serum (FBS) and antibiotic solution (penicillin 100 μg/mL and streptomycin 100 IU/mL), placed in an incubator at a temperature of 36.5–37 °C, in a humidified environment, with 5% CO_2_ [73].

The assessment of the impact on cell viability was carried out by the colorimetric method with 3-(4,5-dimethylthiazol-2-yl)-2,5-diphenyl tetrazolium bromide (MTT), modified after Mosmann [74] and Laville [75], based on the ability of mitochondrial dehydrogenases of living cells to convert the yellow water-soluble substrate (MTT) into dark blue, water-insoluble formazan, the amount of formazan being directly proportional to the number of living cells [76]. The cells were detached with trypsin/EDTA, then counted and resuspended in 96-well microplates (6.5 × 10^3^ cells/well) and maintained at the same temperature and humidity conditions. After monolayer formation (24 h), the cells were treated for 24 and 48 h with the samples added to the final culture medium, the total volume being 300 µL/well. The doses for the liposomes varied in the range of 3.125–50 µg/mL. After treatment, the medium of the cell cultures was replaced with 100 μL of fresh medium, over which 10 μL of a MTT solution (5 mg/mL) was added, followed by incubation at 37 °C for 3 h. After this period, 90 µL was removed and the formazan crystals were dissolved in 50 µL DMSO (Merck KGaA, 64271 Darmstadt, Germany). The absorbance was measured at 570 nm, using the Biochrom EZ Read 400 automatic microplate reader (Biochrom Ltd., Cambridge, UK).

**Statistical analysis**. The results of in vitro assays were expressed as the mean of three parallel assays ± standard error (ES). The difference between mean values for each parameter was expressed using Student’s *t*-test [77].

#### 3.3.4. The Half-Maximal Inhibitory Concentration (IC_50_) Values

The IC_50_ values were calculated using polynomial dose–response curve plots for each liposome tested in the MTT assay.

#### 3.3.5. Cell Morphology Test

The effect of the newly synthesized liposomes was also followed through the prism of the morphological changes induced by them. The morphology of normal V-79-4 cells was observed with a Nikon Eclipse TS 100 (Tokyo, Japan) inverted microscope equipped with an MshOt MS60 digital microscope camera (Guangzhou Micro-shot Technology Co., Ltd., Guangzhou, China). Images were saved as JPEGs [78].

## 4. Conclusions

New cationic liposomes based on DOTAP coated with peptide-functionalized chitosan and loaded with dexamethasone phosphate were obtained by hydration of the thin lipid film, followed by sequential extrusion. TAT and Tet1 peptides were used for the functionalization of the liposomes, which have the role to target specific ear cells and to ensure cellular penetration. Functionalization of CMCS with the two peptides was confirmed by infrared spectroscopy (FTIR) and ^1^H NMR. The mean diameter of the uncovered liposomes was between 167 and 198 nm and depended on the amount of cholesterol in the composition of liposomes. The coating with CMCS led to an increase in the average diameter from 179 to 202 nm. The spherical shape and unilamellar structure of liposomes was revealed by TEM analysis. The in vitro release profiles of dexamethasone from liposomes were determined at 37 °C, in phosphate buffered saline (PBS) at pH 7.4. The release efficiency of dexamethasone phosphate from the uncoated liposomes, compared to the free drug, was greatly slowed down by the lipid matrix. Even at concentration of 100 µg/mL, the liposome suspension did not produce a lysis of erythrocytes greater than 2.5%, after 180 min of incubation. This leads to the conclusion that this type of system can be included in the category of non-hemolytic materials. The in vitro studies on V79-4 normal cell cultures aimed to investigate the degree of toxicity of some newly synthesized liposomes, and morphology studies were carried out to confirm the degree of interference of the nanocarriers with the cells. In conclusion, the obtained liposomes have dimensions below 200 nm, present a spherical shape, a good drug release capacity, are hemocompatible, and non-cytotoxic which makes them suitable as efficient drug delivery systems.

## Figures and Tables

**Table 1:** Hydrodynamic diameter, polydispersity index (PDI), and zeta potential of the obtained liposomes. Data presented as mean ± SD, n = 5.

Sample Code	Lipid Mixture (Weight Ratio)	Hydrodynamic Diameter (nm)	Polydispersity Index (PDI)	Zeta Potential (mV)
**Uncovered liposomes**				
**CLD-1**	EPC/Chol/DOTAP 22/2/1	197 ± 3	0.12 ± 0.02	+11 ± 0.7
**CLD-2**	EPC/Chol/DOTAP 21/2/2	179 ± 1	0.13 ± 0.01	+27 ± 0.9
**CLD-3**	EPC/Chol/DOTAP 19/4/2	167 ± 2	0.14 ± 0.02	+25 ± 2.1
**Liposomes coated with CMCS and CMCS functionalized with peptide mixture**				
**CLDCP-1**	EPC/Chol/DOTAP 22/2/1	202 ± 1	0.06 ± 0.01	−14 ± 0.4
**CLDCP-2**	EPC/Chol/DOTAP 21/2/2	187 ± 1	0.15 ± 0.03	−17 ± 0.1
**CLDCP-3**	EPC/Chol/DOTAP 19/4/2	179 ± 2	0.07 ± 0.01	−18 ± 1.1

**Table 2:** Drug loading and release efficiency. Data presented as mean ± SD, n = 3.

Sample Code	Loading Efficiency (%)	Release Efficiency (%)
**CLD-1**	25 ± 4	37 ± 2
**CLD-2**	23 ± 3	40 ± 4
**CLD-3**	22 ± 3	38 ± 4
**CLDCP-1**	24 ± 2	34 ± 4
**CLDCP-2**	21 ± 2	37 ± 4
**CLDCP-3**	19 ± 2	34 ± 4

**Table 3:** The half-maximal inhibitory concentration (IC50) values after treatment with different new-synthesized cationic liposomes on V79-4 cell line.

Samples	IC_50_ 24 h (µg/mL)	IC_50_ 48 h (µg/mL)
**CL-1**	106.13	61.61
**CL-2**	84.59	31.22
**CL-3**	190.39	37.03
**CLC-1**	77.61	28.91
**CLC-2**	66.75	63.90
**CLC-3**	60.40	35.19
**CLCP-1**	65.46	44.02
**CLCP-2**	84.07	52.29
**CLCP-3**	40.70	29.38

**Table 4:** The experimental program for liposomes obtaining and the samples code.

Sample Code	EPC (mg)	Chol (mg)	DOTAP (mg)	EPC/Chol/DOTAP (Molar Ratio)	Dexamethasone Phosphate (mg)	CMCS + CMCS-P1 + CMCS-P2 * (1%) /Liposomes Suspension (*v*/*v*)
**Uncoated liposomes**						
**CLD-1**	22	2	1	1.00/0.18/0.05	5	-
**CLD-2**	21	2	2	0.95/0.18/0.10		
**CLD-3**	19	4	2	0.86/0.36/0.10		
**Liposomes coated with CMCS + CMCS-P1 + CMCS-P2**						
**CLDCP-1**	22	2	1	1.00/0.18/0.05	5	1/1
**CLDCP-2**	21	2	2	0.95/0.18/0.10		
**CLDCP-3**	19	4	2	0.86/0.36/0.10		

Sample * CMCS/(CMCS-P1+CMCS-P2) = 9/(0.5 + 0.5) (*w*/*w*), CMCS-P1 and CMCS-P2 are carboxylated chitosan functionalized with TAT-NH_2_ and Tet1-NH_2_ peptides.

## Data Availability

The datasets generated during and/or analysed during the current study are available from the corresponding author on reasonable request.

## Supplementary Materials

The following supporting information can be downloaded at: https://www.mdpi.com/article/10.3390/ijms26030922/s1. Figure S1: Structural Characteristics—1H-NMR spectroscopy; Figure S2: The percentage of cumulative drug released from cellulose dialysis tubes with the free dexamethasone phosphate; Figure S3: Hemolysis percentage after 90 and 180 minutes exposure; Figures S4–S12: Morphological aspects of normal V79-4 cells after treatment with liposomes. References [79,80,81,82,83,84] are cited in the Supplementary Materials.
