# Supplementary material for: Double Peptide-Functionalized Carboxymethyl Chitosan-Coated Liposomes Loaded with Dexamethasone as a Potential Strategy for Active Targeting Drug Delivery
Source: Int J Mol Sci. 2025 Jan 22;26(3):922. doi: 10.3390/ijms26030922 (PMC11816442; doi:10.3390/ijms26030922)
Supplement: Supplementary file 1 [file ijms-26-00922-s001.zip › ijms-3385233-supplementary.pdf]

Supplementary material

Double peptide-functionalized carboxymethyl chitosan-coated liposomes loaded with dexamethasone as a potential strategy for active targeting drug delivery

Loredana Iftode[1,2], Anca Niculina Cadinoiu [3*], Delia Mihaela Raţă [3*], Leonard Ionut Atanase [3,4], Gabriela Vochita [5], Luminita Radulescu [1], Marcel Popa [2,3,4*] and Daniela Gherghel [5]

[1] Faculty of Medicine, "Grigore T. Popa", University of Medicine and Pharmacy, Iasi, Romania;
[2] "Cristofor Simionescu" Faculty of Chemical Engineering and Environmental Protection, "Gheorghe Asachi" Technical University, Iasi, Romania;
[3] Department of Biomaterials, Faculty of Medical Dentistry, "Apollonia" University of Iasi, Iasi, Romania
[4] Academy of Romanian Scientists, Bucharest, Romania
[5] NIRDBS - Institute of Biological Research Iasi, Iasi, Romania

* Correspondence: marpopa2001@yahoo.fr (M.P.); jancaniculina@yahoo.com (A.N.C); iureadeliamihaela@yahoo.com (D.M.R.)

Contents

Structural Characteristics - ^{1}H-NMR spectroscopy ……………………………………………………Figure S1

The percentage of cumulative drug released from cellulose dialysis tubes with the free dexamethasone phosphate…………………………………………………………...……………........……………..……Figures S2

Hemolysis percentage after 90 and 180 minutes exposure ………………………………………..Figures S3

Morphological aspects of normal V79-4 cells after treatment with liposomes ………Figure S4 – Figure S12

1. *Structural Characteristics - ^{1}H-NMR spectroscopy*

^{1}H-NMR measurements of CMCS-Tet and CMCS-TAT were performed on a 400 MHz Bruker Neo-1 high-resolution liquid NMR spectrometer (Rheinsteitten, Germany) for direct detection probe, with 5 mm Quadra core probes, QNP (four cores, 1H/13C/19F/29Si). Sample solutions were prepared with deuterated water and 1 N HCl as solvent.

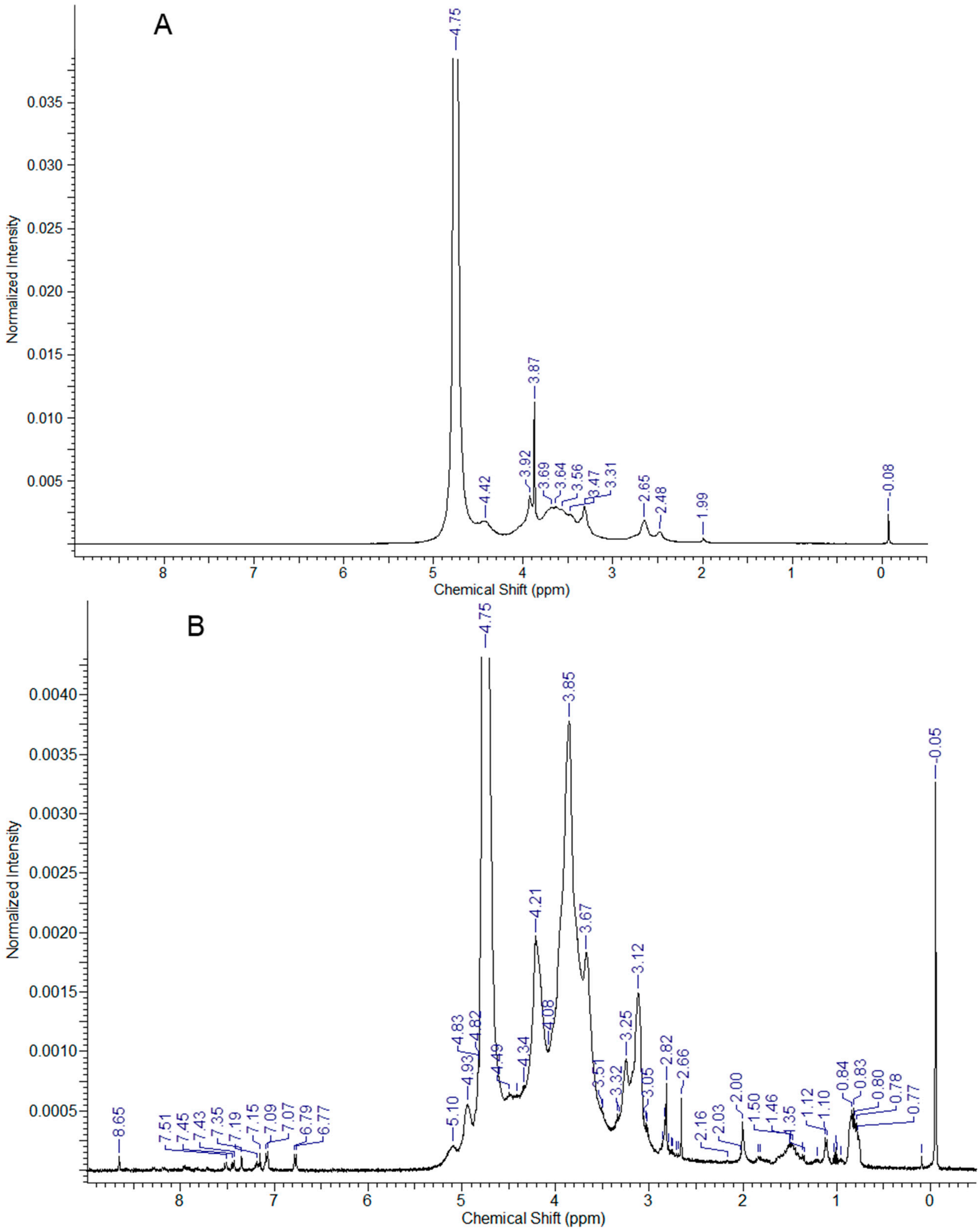

A
Normalized Intensity
Chemical Shift (ppm)
4.75
3.87
4.42
3.92
3.69
3.64
3.56
3.47
3.31
2.65
2.48
1.99
-0.08
B
Normalized Intensity
Chemical Shift (ppm)
8.65
7.51
7.45
7.43
7.35
7.19
7.15
7.09
7.07
6.79
6.77
5.10
4.93
4.83
4.82
4.75
4.49
4.34
4.21
4.08
3.85
3.67
3.51
3.32
3.25
3.12
3.05
2.82
2.66
2.16
2.03
2.00
1.50
1.46
1.35
1.12
1.10
0.84
0.83
0.80
0.78
0.77
-0.05

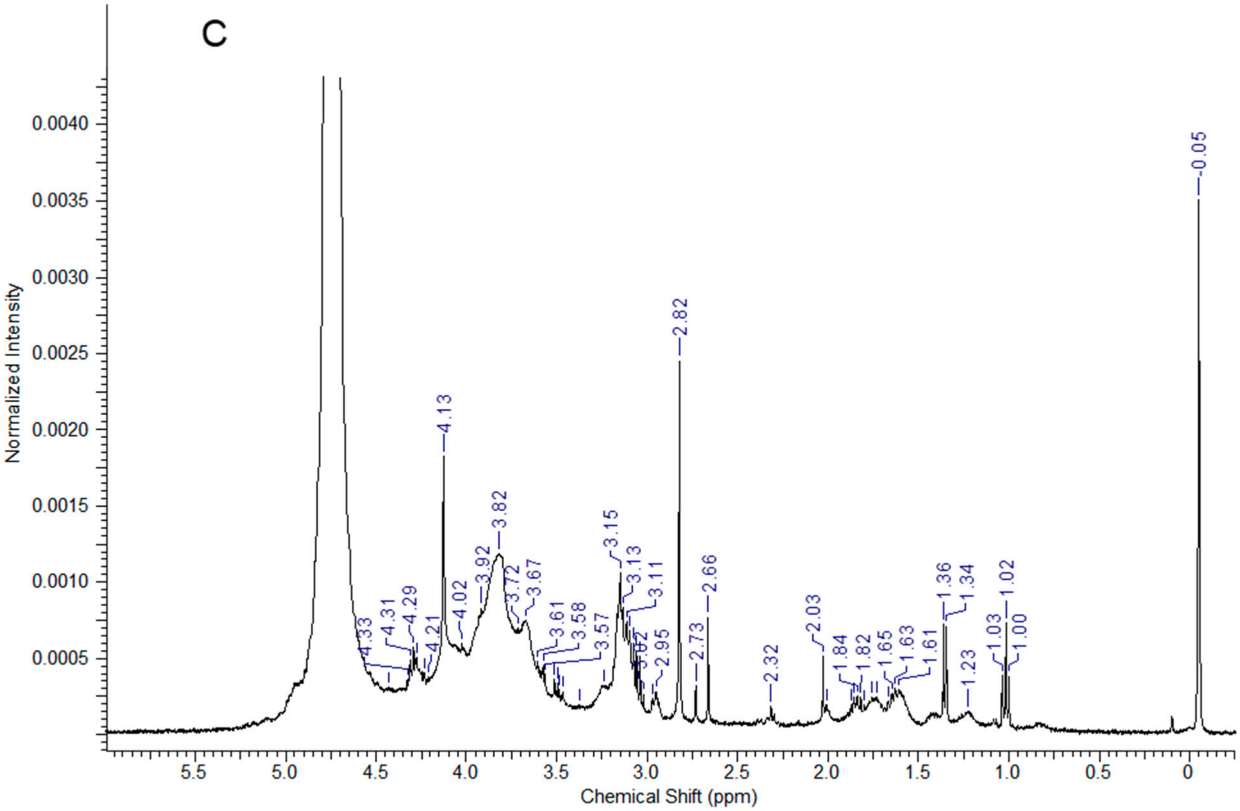

Figure S1. ^{1}H NMR spectra of carboxylated chitosan (A); carboxylated chitosan functionalized with the Tet1 peptide (B) and TAT peptide (C)

Figure S1 shows the ^{1}H NMR spectrum for carboxylated chitosan (A); carboxylated chitosan functionalized with the Tet1 peptide (B) and TAT peptide (C). The ^{1}H NMR spectrum of CMCS (A) shows a peak at approximately 2.068 ppm which can be attributed to the hydrogen of the methyl group of acetamide. The signals at approximately 2.554 ppm and 2.723 ppm correspond to the hydrogen at C2 of the glucosamine unit, respectively. The multiple peaks from 3.39 to 3.95 ppm correspond to the protons at C3, C4, C5, C6 [79]. The appearance of the small signal at approximately 4.51 ppm was due to the appearance of mono-substitution on some of the primary hydroxyl sites of the modified chitosan structure, which indicated that some of the hydroxyl groups were also carboxymethylated [80].

In the spectrum of carboxylated chitosan functionalized with the Tet1 peptide (B) and TAT peptide (C) the signals at 3.1 and 4.3 ppm were ascribed to the protons of –CH_2–COO– substituted on the C2 amino group and the C6 hydroxyl group for CMCS [81].

Two of the amino acids in the Tet1 peptide chain (HOOC-HLNILSTLWKYR-NH2) are easier to identify due to their aromatic structure: tryptophan and histidine (Figure S1-A). The four protons on the ring furthest from the backbone of Tryptophan (W) are usually found between 7.1 and 7.7 ppm [82]. The two C-H ring protons of Histidine (H) have chemical shifts at 7.3 and 8.6 ppm [83].

The amino acids in the TAT peptide chain (HOOC-RKKRRQRRR-NH2) are Arginine (R), Lysine (K) and Glutamine (Q). In the 1H NMR spectrum of CMCS-TAT (Figure S1-B) we can distinguish the -CH_2 groups of arginine, which is an amino acid residue repeated 6 times in the TAT peptide structure, which are

usually found between 1.637 and 1.651 ppm, and the shifts of the -NH and $-NH_2$ groups are shown between 3.20 and 3.76 ppm. [84].

2. *The in vitro drug release*

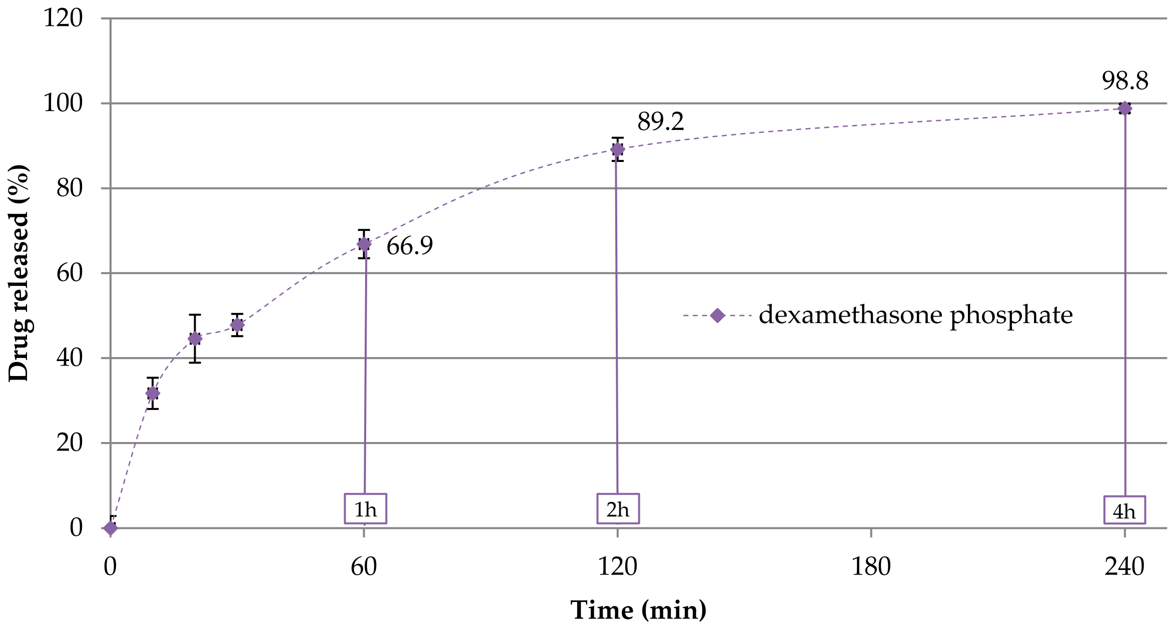

Figure S2. The percentage of cumulative drug released from cellulose dialysis tubes with the free dexamethasone phosphate, highlighting the released percentage at specific time intervals (1h, 2h and 4h). Data presented as mean ± SD, n = 3

3. *The in vitro hemolytic potential*

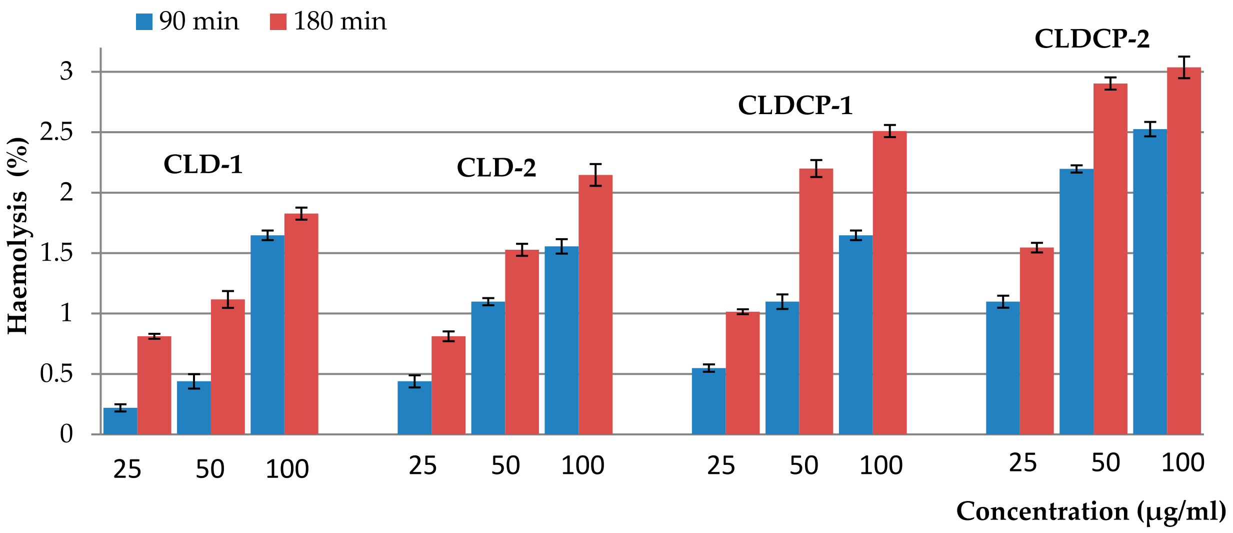

Figure S3. Hemolysis percentage after 90 and 180 minutes exposure

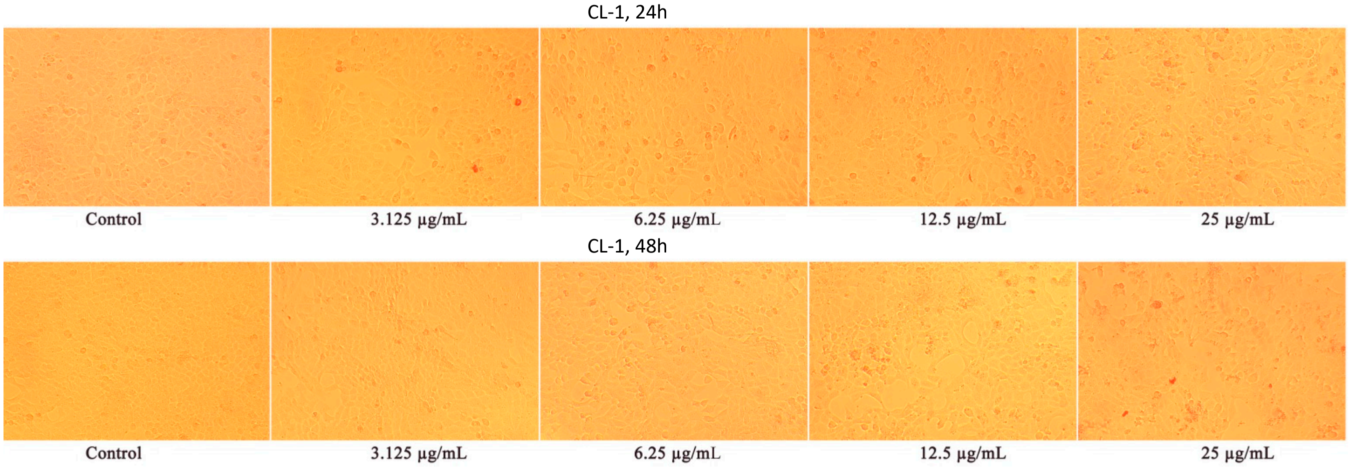

Figure S4. Morphological aspects of normal V79-4 cells after treatment with CL-1 for 24 and 48 hours.

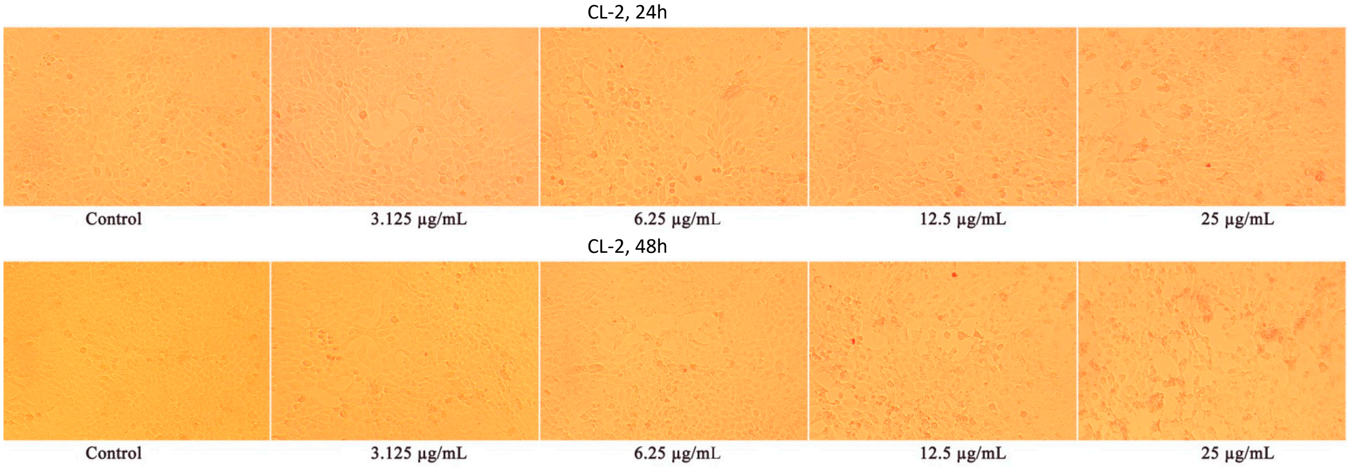

Figure S5. Morphological aspects of normal V79-4 cells after treatment with CL-2 for 24 and 48 hours.

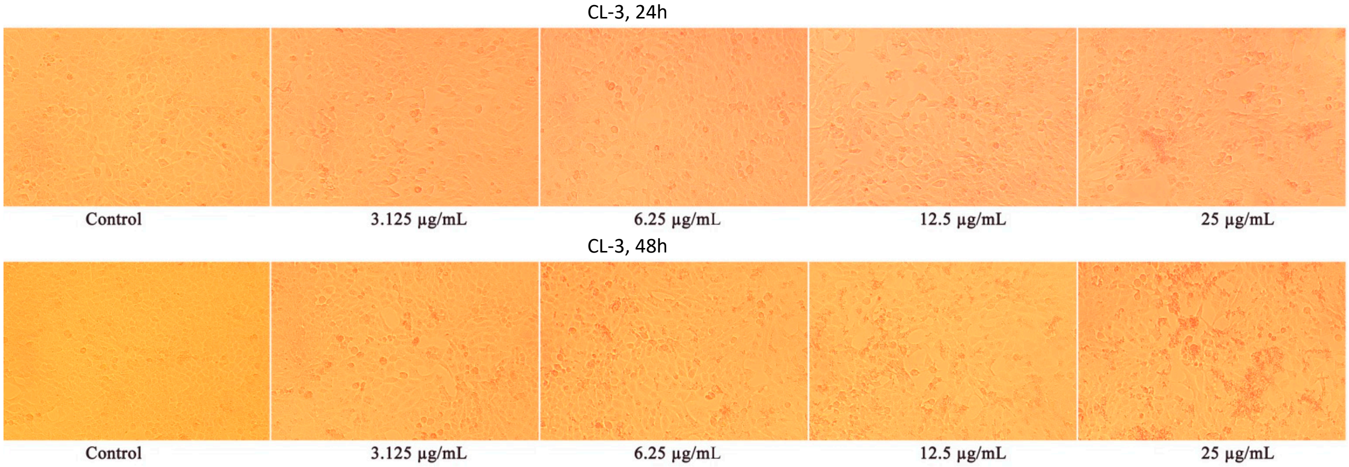

Figure S6. Morphological aspects of normal V79-4 cells after treatment with CL-3 for 24 and 48 hours.

CLC-1, 24h

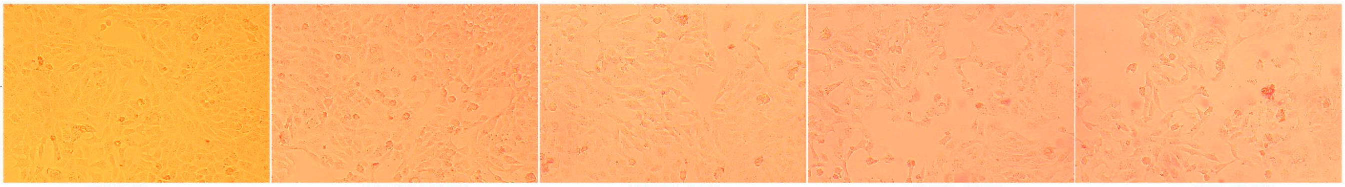

Control 3.125 μg/mL 6.25 μg/mL 12.5 μg/mL 25 μg/mL

CLC-1, 48h

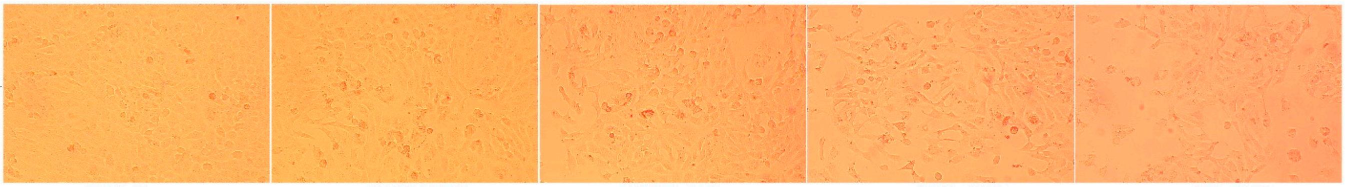

Control 3.125 μg/mL 6.25 μg/mL 12.5 μg/mL 25 μg/mL

Figure S7. Morphological aspects of normal V79-4 cells after treatment with CLC-1 for 24 and 48 hours.

CLC-2, 24h

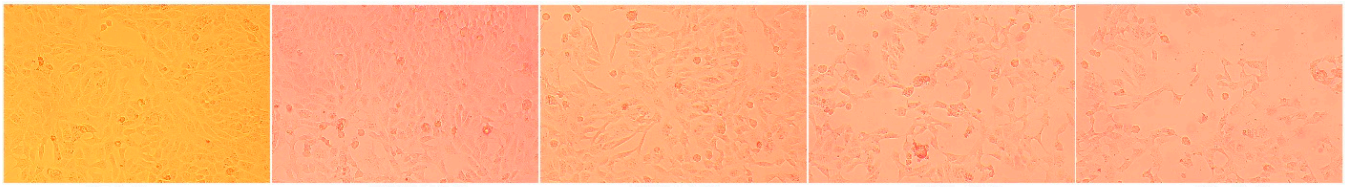

Control 3.125 μg/mL 6.25 μg/mL 12.5 μg/mL 25 μg/mL

CLC-2, 48h

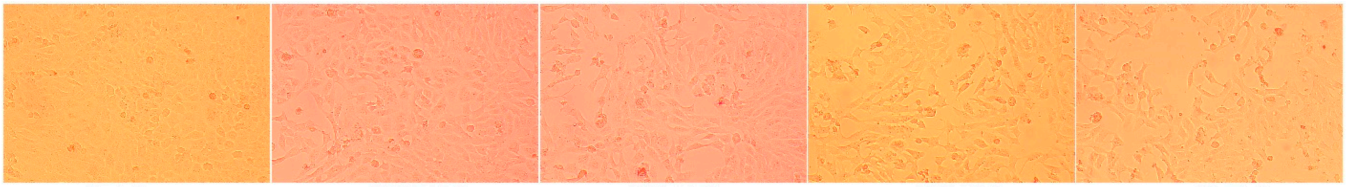

Control 3.125 μg/mL 6.25 μg/mL 12.5 μg/mL 25 μg/mL

Figure S8. Morphological aspects of normal V79-4 cells after treatment with CLC-2 for 24 and 48 hours.

CLC-3, 24h

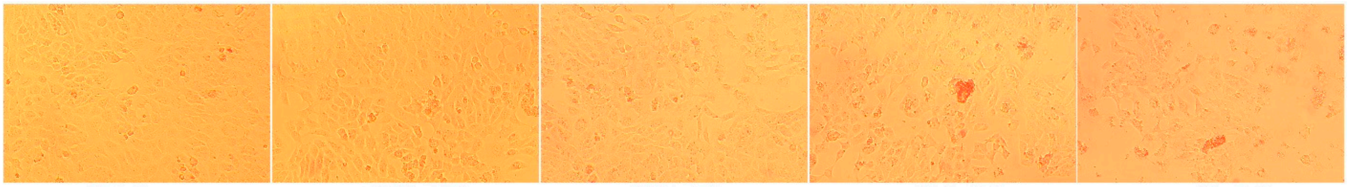

Control 3.125 μg/mL 6.25 μg/mL 12.5 μg/mL 25 μg/mL

CLC-3, 48h

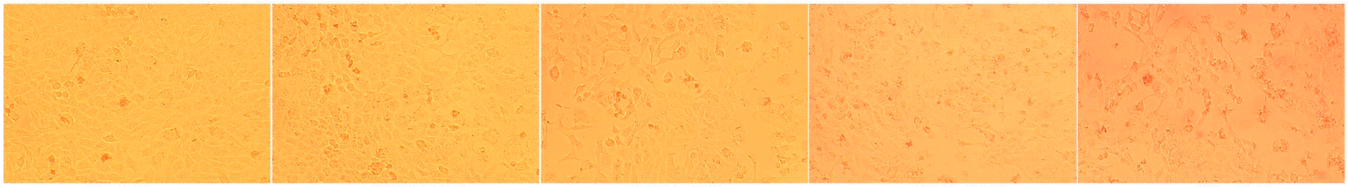

Control 3.125 μg/mL 6.25 μg/mL 12.5 μg/mL 25 μg/mL

Figure S9. Morphological aspects of normal V79-4 cells after treatment with CLC-3 for 24 and 48 hours.

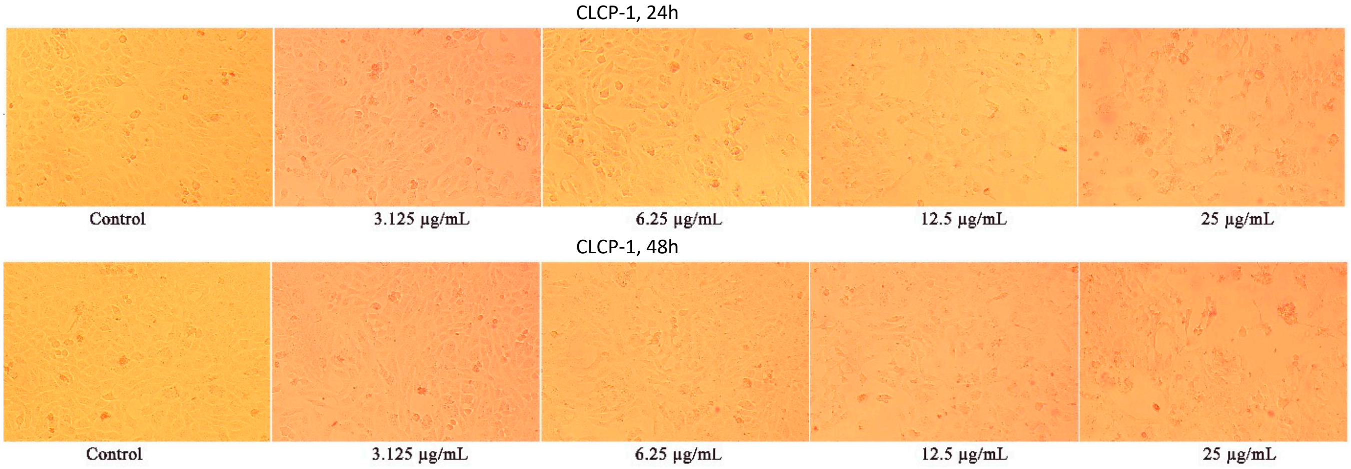

Figure S10. Morphological aspects of normal V79-4 cells after treatment with CLCP-1 for 24 and 48 hours.

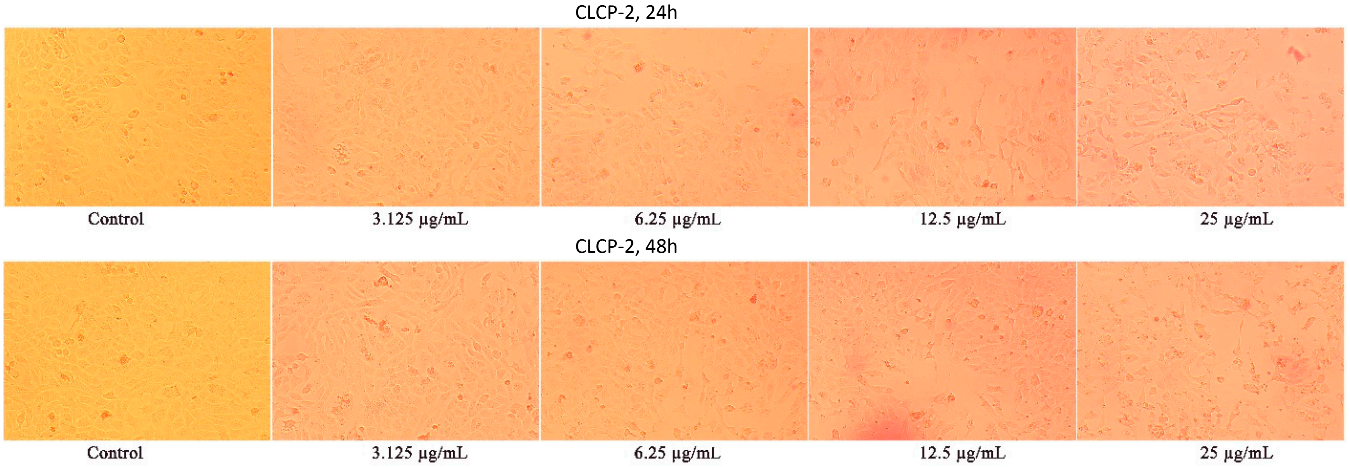

Figure S11. Morphological aspects of normal V79-4 cells after treatment with CLCP-2 for 24 and 48 hours.

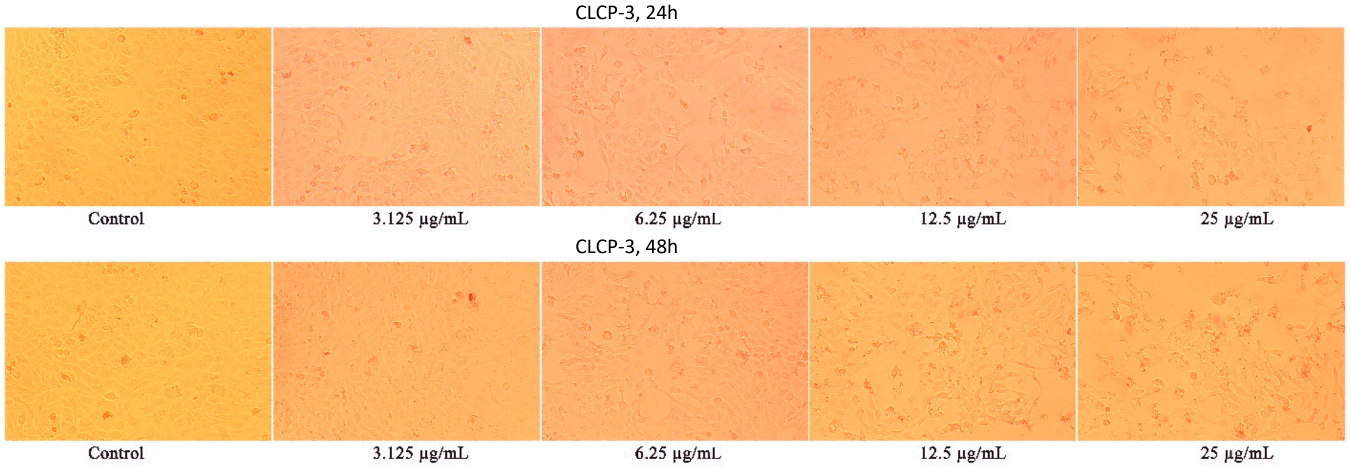

Figure S12. Morphological aspects of normal V79-4 cells after treatment with CLCP-3 for 24 and 48 hours.

References

79. Cerrutti, B.M.; Lamas, J.C.; Campana-Filho, S.P.; Frollini, E. Carboxymethyl Chitosan: Preparation and Use in Colloidal Ceramic Processing. *J. Polym. Environ.* **2012**, *21*, 816–825. https://doi.org/10.1007/s10924-012-0566-7.

80. Vaghani, S.S.; Patel, M.M.; Satish, C.S.; Patel, K.M.; Jivani, N.P. Synthesis and characterization of carboxymethyl chitosan hydrogel: Application as site specific delivery for lercanidipine hydrochloride. *Bull. Mater. Sci*. **2012**, *35*, 1133–1142.. https://doi.org/10.1007/s12034-012-0413-4.

81. Du, J.; Hsieh, Y.L. Nanofibrous membranes from aqueous electrospinning of carboxymethyl chitosan. *Nanotechnology* **2008**, *19*, 125707. https://doi.org/10.1088/0957-4484/19/12/125707.

82. Malta, L.F.; Senra, J.D.; Tinoco, L.W.; Medeiros, M.E.; Antunes, O.A. Chiral Recognition of 2-Hydroxypropyl-alpha-cyclodextrin Towards DLTryptophan. *Lett. Org. Chem.* **2009**, *6*, 258–263. https://doi.org/10.2174/157017809787893091.

83. Tchipilov, T.; Meyer, K.; Weller, M.G. Quantitative 1H Nuclear Magnetic Resonance (qNMR) of Aromatic Amino Acids for Protein Quantification. *Methods Protoc.* **2023**, *6*, 11. https://doi.org/10.3390/mps6010011.

84. Aslan, H.K.; Kuşçulu, N.G. Investigation of chemical activity, SCHIFF base reactions and staining effects of some amino acids by spectrophotometric and theorical methods. *J. Indian Chem. Soc.* **2021**, *99*, 100315. https://doi.org/10.1016/j.jics.2021.100315.
